# Breast cancers with high proliferation and low ER-related signalling have poor prognosis and unique molecular features with implications for therapy

**DOI:** 10.1038/s41416-023-02477-7

**Published:** 2023-11-07

**Authors:** Luca Licata, Marco Barreca, Barbara Galbardi, Matteo Dugo, Giulia Viale, Balàzs Győrffy, Thomas Karn, Lajos Pusztai, Luca Gianni, Maurizio Callari, Giampaolo Bianchini

**Affiliations:** 1 Department of Medical Oncology, San Raffaele Hospital, Milan, Italy; 2 School of Medicine and Surgery, Vita-Salute San Raffaele University, Milan, Italy; 3 University of Milano-Bicocca, Milan, Italy; 4 Department of Bioinformatics, Semmelweis University, Budapest, Hungary; 5 Cancer Biomarker Research Group, Research Centre for Natural Sciences, Budapest, Hungary; 6 Goethe University Hospital Frankfurt, Frankfurt, Germany; 7 Yale Cancer Center, Yale School of Medicine, New Haven, CT USA; 8 Michelangelo Foundation, Milan, Italy

**Keywords:** Translational research, Biomarkers

## Abstract

**Background:**

Luminal breast cancers with high proliferation (MKS^hi^) and low ER-related signalling (ERS^lo^) have a poor prognosis. We investigated treatment responses and molecular features of MKS^hi^/ERS^lo^ tumours to inform potential therapies.

**Methods:**

Gene expression data from patients who received neoadjuvant chemotherapy (NAC) without (MDACC, *N* = 199) or with pembrolizumab (I-SPY2, *N* = 40), or endocrine therapy (NET) without (POETIC, *N* = 172) or with palbociclib (NeoPalAna, *N* = 32) were analyzed to assess treatment response by MKS/ERS-subgroups. TCGA was used to assess the mutational landscape and biomarkers associated with palbociclib-resistance (Cyclin-E, RBsig, IRPR) and immunotherapy-response (TMB, TILs, T-cell inflamed) by MKS/ERS-subgroups.

**Results:**

Compared to MKS^hi^/ERS^hi^ tumours, MKS^hi^/ERS^lo^ tumours had higher pathological response rates to NAC (22% vs 8%, *p* = 0.06) but a higher recurrence risk (4-year metastasis-free survival 70% vs 94%, *p* = 0.01). MKS^hi^/ERS^lo^ tumours frequently harboured TP53 (34%) and PIK3CA (33%) mutations, and showed high expression of Cyclin-E, RBsig and IRPR, high TMB and elevated TIL and T-cell inflamed metagene expression. MKS^hi^/ERS^lo^ tumours retained high proliferation after NET with or without palbociclib but had higher pathological complete response rates when pembrolizumab was added to NAC (42% vs 21%, *p* = 0.07).

**Conclusions:**

MKS^hi^/ERS^lo^ tumours have dismal outcomes and are enriched in chemotherapy-sensitive but ET- and palbociclib-resistant tumours. Biomarker analysis and clinical data suggest a potential role for immunotherapy in this group.

## Introduction

Estrogen receptor-positive, human epidermal growth factor receptor 2-negative (ER+/HER2−) breast cancers are biologically and clinically heterogeneous [1]. Molecular tools that partially capture this heterogeneity are currently utilized in clinical practice to tailor adjuvant treatment [2, 3]. Genomic signatures offer valuable prognostic information and help in estimating adjuvant chemotherapy benefits [4]. Their use has led to treatment de-escalation and spared a significant number of low-risk patients from unnecessary chemotherapy. However, among patients with high genomic risk who are candidates for both chemotherapy and endocrine therapy, many continue to relapse, even with node-negative disease, indicating that further improvements in treatment are needed [5]. Previous studies also suggested that many high genomic-risk cancers also show reduced sensitivity to endocrine therapy [6], underscoring the importance of identifying new therapeutic strategies to improve outcomes.

Previously, we showed that a combination of a proliferation-related gene signature score (MKS) [7] and the estrogen-related gene expression module (ERS) of the Oncotype DX recurrence score [8] can provide prognostic information comparable to commercially available tools [9]. Among highly proliferative ER+/HER2− breast cancers, tumours with low ER-related signalling, hereafter referred to as MKS^hi^/ERS^lo^, exhibit the highest risk of recurrence despite adjuvant endocrine therapy, show poor response to neoadjuvant letrozole endocrine therapy, and account for most of early recurrences during adjuvant tamoxifen treatment [9]. Additionally, breast cancers with high proliferation/low ER-related gene expression have poor long-term survival following chemo-endocrine therapy, even though a subset of these patients experience pathological complete response (pCR) [10].

In this study, we conducted a comprehensive analysis across multiple ER+/HER2− breast cancer cohorts to evaluate the sensitivity of the MKS^hi^/ERS^lo^ subgroup to currently available treatments for ER+/HER2− early breast cancer, including chemotherapy with or without immunotherapy, and endocrine therapy with or without CDK4/6 inhibitors (CDK4/6i). We investigated genomic and transcriptomic differences between the MKS^hi^/ERS^lo^ subgroup and other ER+/HER2− tumours and examined markers that have been reported to predict benefit from CDK4/6i and immune checkpoint inhibitors (ICI). Our findings shed light on the clinical features of these high genomic-risk cancers and suggest novel therapeutic strategies to improve patient survival.

## Methods

### Molecular classification of ER+/HER2− tumours based on MKS and ERS metagenes

Tumour samples were classified according to MKS and ERS as previously described [7]. Briefly, MKS was calculated as the average expression of 12 kinases involved in mitosis, spindle checkpoint, and G2-M transition [7]. ERS was defined as the average expression of the four genes in the estrogen module of the Oncotype DX assay [8] (Table 1). The classification of high or low expression for MKS and ERS metagenes was based on the cohort-specific median value used as a threshold. In Affymetrix datasets, the probe sets reported in Table 1 were included. For expression data obtained from different microarray platforms or RNA-seq, all probes targeting metagene members were subjected to clustering analysis to determine correlations. Probes with a correlation <0.4 in the dendrogram analysis were excluded from calculating the metagene expression level.

**Table 1:** Genes and probe sets used to define the MKS and ERS.

Symbol	Probe set	Description
Mitosis Kinase Score (MKS)		
PLK1	202240_at	polo-like kinase 1 (Drosophila)
CDK1	203213_at	cyclin-dependent kinase 1
BUB1B	203755_at	budding uninhibited by benzimidazoles 1 homolog beta (yeast)
NEK2	204641_at	NIMA (never in mitosis gene a)-related kinase 2
TTK	204822_at	TTK protein kinase
MELK	204825_at	maternal embryonic leucine zipper kinase
PLK4	204887_s_at	polo-like kinase 4 (Drosophila)
CHEK1	205394_at	CHK1 checkpoint homolog (S. pombe)
AURKA	208079_s_at	aurora kinase A
AURKB	209464_at	aurora kinase B
BUB1	209642_at	budding uninhibited by benzimidazoles 1 homolog (yeast)
PBK	219148_at	PDZ binding kinase
Estrogen-related score (ERS)		
BCL2	203685_at	B-cell CLL/lymphoma 2
ESR1	205225_at	estrogen receptor 1
PGR	208305_at	progesterone receptor
SCUBE2	219197_s_at	signal peptide, CUB domain, EGF-like 2

### Datasets

#### MD Anderson dataset

Normalized gene expression data were downloaded from the GEO repository (http://www.ncbi.nlm.nih.gov/geo/, GSE25066) for 298 patients with stage II–III ER+/HER2− (gene-based receptor assignment) breast cancer treated with taxane-anthracycline-based neoadjuvant chemotherapy followed by adjuvant endocrine therapy after surgery. Detailed information regarding patients’ characteristics and treatment has been reported elsewhere [11]. The primary endpoints were pathological response to neoadjuvant chemotherapy and distant event-free survival (DEFS). Pathological response was defined using the residual cancer burden (RCB) categories [12]. We identified two response groups; the pathR group, including pCR (RCB 0) and minimal residual cancer (RCB I) cases; and the RD group, including moderate (RCB-II) or extensive (RCB-III) residual disease [12]. Distant event-free survival was defined as the interval from the initial diagnostic biopsy to the occurrence of distant metastases or death from any cause. Based on the predefined cut-off points for the entire cohort [13], we assigned the cases into 4 molecular subgroups based on MKS and ERS distribution; MKS^lo^/ERS^hi^ (*n* = 37), MKS^lo^/ERS^lo^ (*n* = 62), MKS^hi^/ERS^hi^ (*n* = 68), MKS^hi^/ERS^lo^ (*n* = 131).

#### POETIC dataset

Gene expression profiling and clinical data from the aromatase inhibitor (AI)-treated cohort of the POETIC trial [14] were obtained from the GEO repository (GSE105777 and GSE126870). In POETIC, postmenopausal patients with ER+ breast cancer were randomly assigned to receive perioperative AI or no treatment, starting approximately 14 days before surgery and continuing for 14 days after surgery. Additional sample annotation from this trial was obtained from Gao et al. [15]. The efficacy endpoint was proliferation arrest captured as the percentage of Ki67-positive cells by immunohistochemistry (IHC) after two weeks of preoperative AI treatment. Post-treatment Ki67 levels below 10% were considered a response to endocrine therapy and surrogate for long-term benefit [14]. In this dataset, we confirmed a strong correlation between the Ki67 IHC per cent positivity and the MKS metagene expression level. We assigned the 172 ER+/HER2− tumours into 4 molecular subgroups based on MKS and ERS distribution; MKS^lo^/ERS^hi^ (*n* = 43); MKS^lo^/ERS^lo^ (*n* = 43), MKS^hi^/ERS^hi^ (*n* = 43), MKS^hi^/ERS^lo^ (*n* = 43).

#### NeoPalAna dataset

Normalized gene expression profiles and sample annotation of the NeoPalAna cohort [16] were obtained from GEO (GSE93204). Missing data were imputed using the lowest expression value in the matrix. NeoPalAna was a single-arm phase II trial that enrolled 50 patients with ER+/HER2− breast cancer to receive anastrozole for 4 weeks (cycle 0) followed by adding palbociclib (C1D1) for up to four 28-day cycles [16]. MKS and ERS expression was evaluated in the 32 patients who had available samples at baseline before treatment. Samples were assigned into 4 molecular subgroups; MKS^lo^/ERS^hi^ (*n* = 10); MKS^lo^/ERS^lo^ (*n* = 6); MKS^hi^/ERS^hi^ *n* = 6); MKS^hi^/ERS^lo^ (*n* = 10). MKS was also calculated at cycle 1 day 15 (C1D15) for 23 samples and post-treatment subgroups were also created; MKS^lo^/ERS^hi^ (*n* = 9); MKS^lo^/ERS^lo^ (*n* = 3); MKS^hi^/ERS^hi^ (*n* = 5); MKS^hi^/ERS^lo^ (*n* = 6).

#### I-SPY2 dataset

Gene expression profiling and clinical data of patients enrolled in the I-SPY2 trial and treated on the pembrolizumab arm of the trial were obtained from the GEO database (GSE194040). In this treatment arm, women with HER2-negative breast cancer were randomly assigned to receive neoadjuvant weekly paclitaxel with (*n* = 40) or without (*n* = 94) pembrolizumab, followed by AC chemotherapy and surgery [17]. All hormone receptor-positive (HR+) cancers were MammaPrint high-risk. Given that the genes included in the MammaPrint assay are primarily associated with proliferation and metastatic processes [18], we expected that all HR+ patients in this trial had tumours with high proliferation. Consequently, we used only the median ERS value as a threshold to stratify patients into ERS^hi^ (*n* = 51 paclitaxel arm; *n* = 16 paclitaxel + pembrolizumab arm) and ERS^lo^ (*n* = 43 paclitaxel arm; *n* = 24 paclitaxel + pembrolizumab arm) categories. The efficacy endpoint was pCR rate in the two ERS categories in both treatment arms.

#### TCGA dataset

Whole-exome sequencing (WES), RNA sequencing (RNA-seq) and clinical information of the TCGA breast cancer cohort were obtained from the TCGA public access portal (http://cancergenome.nih.gov/) (accessed on 22 September 2021). Individual patient files were merged into a single database using the TCGAbiolinks R package [19]. Receptor status was assigned based on ER and HER2 determined by routine pathology. For samples with missing ER and HER2 status, we assigned receptor status based on mRNA expression of ESR1 and ERBB2 genes as previously described [7, 20]. ER positivity was defined as log2 FPKM > 2.145, and HER2 positivity as log2 FPKM > 6.32. MKS and ERS median values were defined within all ER+ tumours (*n* = 852). For our analysis, we selected only tumours with both WES and RNA-seq data available including 640 ER+/HER2−, 174 triple-negative (TNBC), and 158 HER2+ cancers. The 640 ER+/HER2− tumours were then classified into four MKS/ESR subgroups: MKS^lo^/ERS^hi^ (*n* = 209), MKS^lo^/ERS^lo^, (*n* = 129), MKS^hi^/ERS^hi^ (*n* = 146), MKS^hi^/ERS^lo^ (*n* = 156).

### Genomic and transcriptomic analyses

WES data from TCGA were used to compare the mutational landscape among the four MKS/ERS subgroups of ER+/HER2− breast cancers. Since targeting rare mutations in the early setting could be challenging, the top 15 most frequently mutated genes were included in the analysis. Frequently mutated genes that are not considered to be driver genes were also excluded [21]. The tumour mutational burden (TMB) was computed as the number of synonymous and non-synonymous mutations in the whole exome [22].

RNA-seq data were used to assess the expression of candidate predictive biomarkers of response to CDK4/6 inhibition including the Rb1 loss-of-function signature (RBsig) [23], the Interferon-Related Palbociclib-Resistance Signature (IRPS) [24] and expression of *CCNE1* (cyclin E) gene [25]. We also assessed the expression of an immune metagene associated with T cell infiltration (TILs metagene) [26], and the T cell–inflamed gene expression signature (T cell–inflamed GEP) that is predictive of response to immune-checkpoint blockade [27].

### Statistical analysis

Statistical analyses were performed using R software v3.5.3 (R Development Core Team, Vienna, Austria). Significance was based on *P* < 0.05 and 95% confidence interval (CI) estimates.

Biomarker expression among molecular groups was compared using a two-sided Student’s t-test. The frequency distribution of mutations among groups was compared using the Cochran-Mantel-Haenszel χ2 test. Survival analysis was performed using the Kaplan-Meier method and the difference among groups was estimated by log-rank test. The Cox proportional hazard model (univariate analysis) was used to estimate hazard ratios (HRs) and corresponding 95% CIs. In the evaluation of pCR rates, the Odds ratio and 95% CI were evaluated through the median-unbiased estimation method.

## Results

### Among highly proliferative ER+/HER2− breast cancers, ER-related gene expression influences response to neoadjuvant chemotherapy and prognosis

Among ER+/HER2− breast cancers, highly proliferative tumours are considered primary candidates for systemic chemotherapy in addition to adjuvant endocrine therapy. In the MDACC dataset, we examined pathR and metastases-free survival (DEFS, defined as the time from randomization to the development of any distant metastasis or death) by MKS/ERS categories. Among 298 ER+/HER2− breast cancers, 199 were classified as MKS^hi^ [11]. Out of these, 68 were MKS^hi^/ERS^hi^ and 131 were MKS^hi^/ERS^lo^. The median follow-up was 3.1 years. Patients’ baseline characteristics were reported in Supplementary Table 1.

**Fig. 1:**
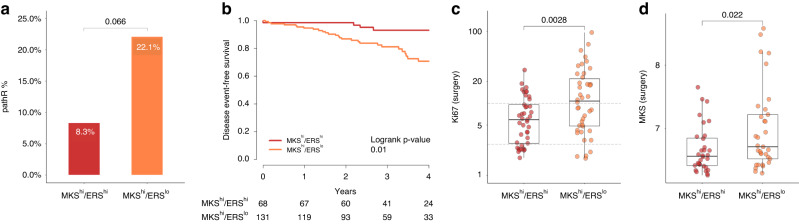
Clinical and pathological outcomes of patients with MKS^hi^ tumours treated with neoadjuvant systemic therapy. **a** PathR rate in patients with MKS ^**hi**^/ERS ^**hi**^ and MKS ^**hi**^ /ERS ^**lo**^ tumours treated with taxane-anthracycline-based neoadjuvant chemotherapy at MD Anderson Cancer Centre. **b** 4-year distant event-free survival in patients with MKS ^**hi**^/ERS ^**hi**^ and MKS ^**hi**^/ERS ^**lo**^ treated with taxane-anthracycline-based neoadjuvant chemotherapy at MD Anderson Cancer Centre. **c**, **d** Ki67 levels (**c**) and MKS expression (**d**) at the surgery in patients with MKS ^**hi**^/ERS ^**hi**^ and MKS ^**hi**^/ERS ^**lo**^ treated with neoadjuvant AI in the POETIC study.

The pathR rate was numerically higher in the MKS^hi^/ERS^lo^ group compared to the MKS^hi^/ERS^hi^ group (22% vs 8%, *p* = 0.06) (Fig. 1A). Importantly, the pathR rates in MKS^hi^/ERS^hi^ cancers were similar to the those in the MKS^lo^ cancers, and within the MKS^lo^ group, ERS had no impact on pathR rates (Supplementary Fig. S1A). Overall, the MKS^hi^/ERS^lo^ group had a significantly worse 4-year DEFS (70% vs 94% in MKS^hi^/ERS^lo^ and MKS^hi^/ERS^hi^ groups, respectively; Logrank *p* = 0.01) (Fig. 1B, and Supplementary Fig. S1B), driven by the poor outcome of those with residual disease (RD). All distant recurrences during the first 2 years of adjuvant endocrine therapy occurred in the MKS^hi^/ERS^lo^ group. Among patients with MKS^hi^/ERS^hi^ tumours, the risk of distant recurrence in the RD and pathR groups were similar (4-year DEFS 93% and 100% respectively; *p* = 0.6). In contrast, among patients with MKS^hi^/ERS^lo^ tumours, those with RD had a significantly worse outcome than those with pathR (4-year DEFS 61% and 100%, respectively; HR 9.1; 95% CI: 1.23–67.4; *p* = 0.005) (Supplementary Fig. S1C, D). These findings indicate that the MKS^hi^/ERS^lo^ cancers represent the most chemotherapy-sensitive subset of ER+/HER2− cancers, but if they fail to achieve a pathR, their prognosis is poor. The prognostic value of RD varies by ERS status, with 4-year DEFS of 93% in MKS^hi^/ERS^hi^ compared to 61% in MKS^hi^/ERS^lo^ cancers (*p* = 0.0005).

### Highly proliferative ER+/HER2− breast cancers with low ER-related gene expression have lower proliferation suppression after neoadjuvant endocrine therapy

In the neoadjuvant endocrine therapy dataset (POETIC [14]) we assessed proliferation suppression after two weeks of AI therapy in the different MKS/ERS subgroups. The MKS^hi^/ERS^lo^ tumours exhibited significantly higher Ki67 levels compared to MKS^hi^/ERS^hi^ tumours at surgery (*p* = 0.0028, Fig. 1c). Among the MKS^hi^/ERS^lo^ cancers, 21 out of 42 (50%) had Ki67 > 10% at surgery, whereas only 10 out of 41 (24.4%) MKS^hi^/ERS^hi^ cancers showed similar high Ki67 levels, indicating lower responsiveness to endocrine therapy in MKS^hi^/ERS^lo^ cancers. We also assessed the post-treatment proliferation suppression after two weeks using the MKS signature itself. Consistent with the Ki67 IHC results, the MKS expression levels were significantly higher in the MKS^hi^/ERS^lo^ compared to MKS^hi^/ERS^hi^ cancers (*p* = 0.022, Fig. 1d and Supplementary Fig. S1E, F).

### Clinical, genomic, and transcriptomic features of the MKS^hi^/ERS^lo^ tumours in the TCGA dataset

#### Survival analysis

**Fig. 2:**
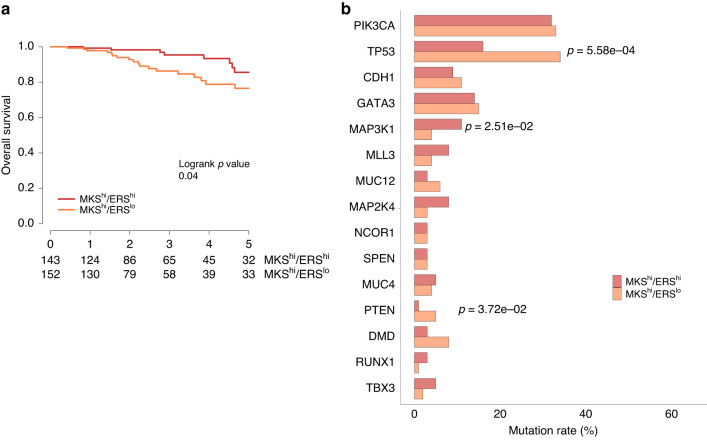
Clinical outcomes and genomic alterations of MKS^hi^ tumours in TCGA. **a** 5-year Overall Survival in patients with MKS^hi^/ERS^hi^ and MKS^hi^/ERS^lo^ tumours. **b** Most frequently mutated genes in MKS^hi^/ERS^hi^ and MKS^hi^/ERS^lo^ tumours.

First, we assessed the prognosis of MKS^hi^/ERS^lo^ tumours using data from the TCGA. We found that the 5-year OS rates were 86% in MKS^hi^/ERS^hi^ tumours (*n* = 143) and 77% in MKS^hi^/ERS^lo^ tumours (*n* = 152) (HR: 0.42; 95% CI: 0.18–0.97; *p* = 0.04) (Fig. 2a). We then extended the survival analysis to include also MKS^lo^/ERS^lo^ (*n* = 125) and MKS^lo^/ERS^hi^ (*n* = 208) tumours. The MKS^hi^/ERS^lo^ tumours retained the poorest survival among all ER+/HER2− breast cancers (*p* = 0.008, Supplementary Fig. S2A). These findings confirm the observation made in the MDACC dataset and define a particularly poor prognosis subset of ER+/HER2− breast cancers that need further improvements in therapy.

#### Mutational landscape by MKS/ERS subgroups

To identify potentially therapeutically targetable genomic alterations, we examined genomic and transcriptomic differences between the MKS^hi^/ERS^lo^ subgroup and other ER+/HER2− tumours. We report the top 15 most frequently mutated genes in ER+/HER2− tumours in the different MKS/ERS subsets (Supplementary Table 2). The MKS^hi^/ERS^lo^ cancers had significantly higher mutation rates in *TP53* and *PTEN*, and a lower mutation rate in *MAP3K1* compared to MKS^hi^/ERS^lo^ cancers (Fig. 2b). To gain a more comprehensive understanding of the mutational landscape across the MKS/ERS subgroups, we expanded our analysis to MKS^lo^/ERS^hi^ and MKS^lo^/ERS^lo^ tumours. Among the four subgroups, we identified 5 genes that were significantly differentially mutated: *PIK3CA, TP53, CDH1, MAP3K1* and *MLL3* (Supplementary Fig. S2B). The most frequently mutated gene overall was *PIK3CA* (39%), with a significantly higher mutation rate in MKS^lo^/ERS^hi^ tumours compared to the three other MKS/ERS subgroups (52% vs 32–33%, *p* = 6.47 × 10^−5^, Supplementary Table 2). *TP53* mutations were significantly more prevalent in MKS^hi^/ERS^lo^ tumours (34%) than in other subgroups (6%, 12% and 16% in MKS^lo^/ERS^hi^, MKS^lo^/ERS^lo^ and MKS^hi^/ERS^hi^, respectively) (*p* = 8.28 × 10^−11^) (Supplementary Table 2). Mutations typically associated with an indolent ER+/luminal A phenotype, such as *CDH1* and *MAP3K1* [28], were more frequent in MKS^lo^/ERS^hi^ tumours, consistent with previous data [28, 29].

#### CDK4/6 inhibitors response marker expression by MKS/ERS subgroup

**Fig. 3:**
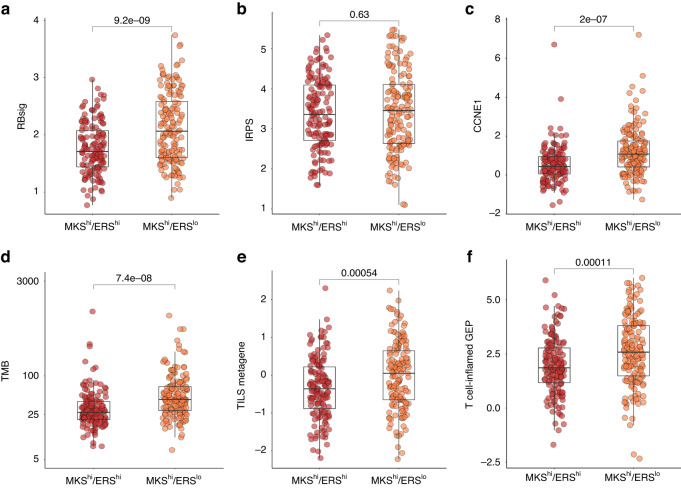
Genomic and transcriptomic features of MKS^hi^ tumours in TCGA dataset. **a** RB signature expression levels. **b** Interferon-Related Palbociclib- Resistance Signature expression levels. **c** Cyclin E expression levels. **d** Tumour mutational burden. **e** T cell infiltration signature expression levels (TILs metagene, by Rody et al.). **f** T cell–inflamed GEP expression levels (by Ayers et al.).

To assess the potential CDK4/6i sensitivity of MKS^hi^/ERS^lo^ tumours, we examined the Rb loss-of-function signature (RBsig), which consists of E2F-associated genes and has been linked to poor prognosis and resistance to palbociclib [23]. The RBsig expression levels were significantly higher in MKS^hi^/ERS^lo^ tumours compared to MKS^hi^/ERS^hi^ tumours (*p* = 9.2 × 10^−9^, Fig. 3a). The MKS^hi^ cancers generally showed significantly higher RBsig expression compared to MKS^lo^/ERS^lo^ and MKS^lo^/ERS^hi^ tumours (*p* = <2.2 × 10^−16^) (Supplementary Fig. S3A).

Next, we evaluated another potential CDK4/6i resistance signature, the IFN-Related Palbociclib-Resistance Signature (IRPS). The IRPS includes 35 genes INF-γ and INF-α regulated genes and was shown to predict CDK4/6i resistance in vitro and in vivo [24]. We found no significant difference in IRPS expression between MKS^hi^/ERS^hi^ and MKS^hi^/ERS^lo^ tumours (*p* = 0.63, Fig. 3b). However, both MKS^hi^ subgroups exhibited significantly higher IRPS expression than MKS^lo^/ERS^hi^ tumours (*p* = <0.01, Supplementary Fig. S3B).

High *CCNE1* mRNA levels, that code for the Cyclin E protein [30], were also linked with poor benefit from the addition of CDK4/6i to endocrine therapy [25]. The MKS^hi^/ERS^lo^ tumours had significantly higher *CCNE1* mRNA expression than MKS^hi^/ERS^hi^ tumours (*p* = 2.0 × 10^−7^) (Fig. 3c), and exhibited the highest *CCNE1* mRNA expression among all ER+/HER2− tumours (Supplementary Fig. S3C). These data raise the possibility that MKS^hi^/ERS^lo^ tumours might derive less benefit from CDK4/6i than other subtypes of ER+/HER2− breast cancers.

#### Immunotherapy response marker expression by MKS/ERS subgroup

A series of reports from the I-SPY neoadjuvant trial has shown improvement in pCR (RCB0) rates by adding ICI to paclitaxel in the MammaPrint-high subset of ER+/HER2− cancers [17, 31]. Therefore, we assessed potential immunotherapy response markers expression in the MKS/ERS subgroups. High TMB has been associated with benefits from ICI in various cancer types [32]. We observed significantly higher median TMB in MKS^hi^/ERS^lo^ compared to MKS^hi^/ERS^hi^ tumours (43 vs 27 synonymous and non-synonymous mutations in the whole exome [mut], *p* = 7.4 × 10^−8^) (Fig. 3d). We also evaluated TMB in MKS^lo^ ER+/HER2− tumours and in HER2+ and TNBC. Among all ER+/HER2− tumours, the MKS^hi^/ERS^lo^ subgroup consistently showed the highest TMB (Supplementary Fig. S4A). Across the entire breast cancer cohort, the median TMBs were 27.0 mut in ER+/HER2−, 37.5 mut in HER2+/ER+, 40.0 mut in HER2+/ER− and 49.5 mut in TNBC (Supplementary Fig. S4B). Notably, TMB was similar between MKS^hi^/ERS^lo^ ER+/HER2− tumours and TNBC (*p* = 0.68).

TMB was inversely correlated with ERS in all ER+/HER2− tumours (Supplementary Fig. S4C) and also in MKS^hi^ tumours (Supplementary Fig. S4D). Furthermore, we investigated whether TMB differed between tumours with or without *PIK3CA* and *TP53* mutations in ER+/HER2− breast cancer. TMB was similar between PIK3CA-wild type and PIK3CA-mutated tumours in the entire cohort and within each MKS/ERS subgroup (Supplementary Fig. S4E). However, TP53-mutated tumours exhibited significantly higher TMB in the entire cohort (*p* = 5.1 × 10^−11^) and in the MKS/ERS subgroups, except for MKS^hi^/ERS^lo^, where TMB was similar between TP53-mutated and TP53-wild type tumours (Supplementary Fig. S4F).

Another biomarker associated with higher ICI response is the infiltration of T cells in the tumour, which can be quantified by the TILs metagene consisting of T cell-related genes [26]. MKS^hi^/ERS^lo^ tumours exhibited significantly higher expression of the TILs metagene compared to MKS^hi^/ERS^hi^ tumours (*p* = 5.4 × 10^−4^) (Fig. 3e). A higher expression of the TILs metagene was also observed in the MKS^lo^/ERS^lo^ compared to MKS^lo^/ERS^hi^ cancers (Supplementary Fig. S5A), indicating an inverse correlation between TILs and the ERS metagene (Supplementary Fig. S5B, C).

Lastly, we investigated the T cell-inflamed GEP, a gene signature composed of IFN-γ- and T cell-related genes, which has been shown to predict response to pembrolizumab across tumour types [27]. Within the MKS^hi^ group, MKS^hi^/ERS^lo^ tumours showed significantly higher expression of the T cell–inflamed GEP compared to MKS^hi^/ERS^hi^ tumours (*p* = 1.1 × 10^−4^) (Fig. 3f). Within the MKS^lo^ group, MKS^lo^/ERS^lo^ tumours had higher expression of the T cell–inflamed metagene than MKS^lo^/ERS^hi^ tumours (Supplementary Fig. S5D). This negative correlation with ERS was also observed across all ER+/HER2− (Supplementary Fig. S5E) and specifically in MKS^hi^ tumours (Supplementary Fig. S5F). Overall, these results consistently demonstrate that the MKS^hi^/ERS^lo^ tumours have high expression of immune markers that predict potential sensitivity to ICI therapy.

### Clinical outcomes in patients receiving neoadjuvant AI plus palbociclib

**Fig. 4:**
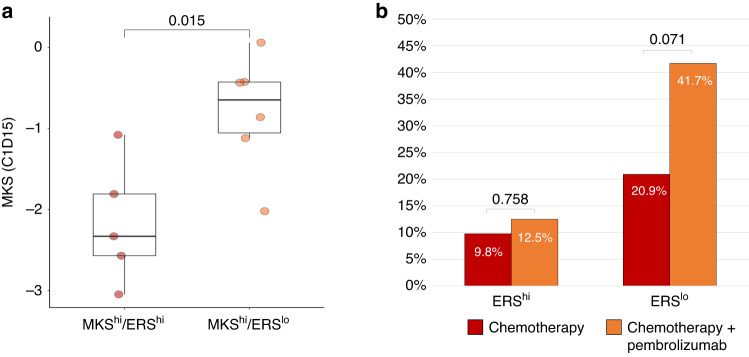
Clinical outcomes in patients receiving neoadjuvant AI plus palbociclib and in patients receiving neoadjuvant chemotherapy with or without pembrolizumab. **a** MKS expression at C1D15 in patients with MKS^hi^/ERS^hi^ and MKS^hi^/ERS^lo^ tumours treated with neoadjuvant AI + palbociclib in the NeoPalAna study (**b**) Pathological complete response rates in patients with hormone receptor-positive tumours treated with taxane- and anthracycline-based neoadjuvant chemotherapy in the I-SPY2 trial (*n* = 134), stratified according to ERS expression.

To evaluate the extent of proliferation suppression with neoadjuvant CDK4/6i plus endocrine therapy in the MKS/ERS subgroups, we analyzed proliferation metrics in C1D15 samples obtained from the NeoPalAna trial, which enrolled patients receiving neoadjuvant anastrozole and palbociclib. After 14 days of preoperative CDK4/6i and endocrine therapy, the expression of the MKS metagene was significantly higher in MKS^hi^/ERS^lo^ compared to MKS^hi^/ERS^hi^ tumours (*p* = 0.015, Fig. 4a). This observation indicates lower treatment sensitivity to the combination therapy in MKS^hi^/ERS^lo^ cancers, which is consistent with the high expression of CDK4/6i resistance markers observed in this subgroup.

### Clinical outcomes in patients receiving neoadjuvant chemotherapy with or without pembrolizumab

Publicly available gene expression data of ER+/HER2− breast cancers from the pembrolizumab arm and corresponding controls of the I-SPY2 trial [17] enabled us to asses the pathologic response rate to ICI therapy based on MKS/ERS classification. All of these tumours were MammaPrint high risk, indicating high proliferation and assumed MKS^hi^. Among these, 24 (60%) and 43 (48%) were classified as ERS^lo^ in the pembrolizumab plus chemotherapy and chemotherapy alone arms, respectively. Within the ERS^hi^ group, the pCR rates were similar between the treatment arms, 9.8% in the chemotherapy alone group and 12.5% in the chemotherapy plus pembrolizumab group (odds ratio: 1.36; 95% CI: 0.16–7.44; *p* = 0.758) (Fig. 4b). In contrast, within the ERS^lo^ group, the addition of pembrolizumab to paclitaxel resulted in a doubling of the pCR rate, from 20.9% in the chemotherapy alone group to 41.7% in the chemotherapy plus pembrolizumab group (odds ratio: 2.65; 95% CI: 0.88–8.22; *p* = 0.071) (Fig. 4b). The *P* value for the interaction test between ERS and treatment arm (chemotherapy plus pembrolizumab) was 0.494.

## Discussion

Proliferation and ER-related signalling recapitulate most of the prognostic heterogeneity of ER+/HER2− breast cancer. Since these two variables provide independent prognostic and chemotherapy and endocrine therapy predictive information [8], taking into account their interactions could improve prognostic and predictive categorization. Previous studies have demonstrated that among highly proliferative ER+/HER2− breast cancers, tumours with low ER-related signalling (i.e. MKS^hi^/ERS^lo^) exhibit poor response rates to neoadjuvant AI and high rates of early relapse during adjuvant tamoxifen, indicating an enrichment of primary endocrine-resistant tumours in this group [9].

In this study, using gene expression and clinical data from the POETIC trial [14], we confirmed in an independent dataset that MKS^hi^/ERS^lo^ tumours respond poorly to neoadjuvant endocrine therapy, as evidenced by the persistently high Ki67 values after neoadjuvant AI therapy. We also showed that MKS^hi^/ERS^lo^ tumours have a high risk of relapse even when treated with neoadjuvant chemotherapy followed by adjuvant endocrine therapy. Although approximately 20% of these cancers achieve a pCR with neoadjuvant chemotherapy, which is associated with excellent recurrence-free survival, those with RD have a high risk of recurrence despite subsequent adjuvant endocrine therapy. The relative resistance of MKS^hi^/ERS^lo^ tumours to endocrine therapy is further supported by the notable difference in the 4-year DEFS between MKS^hi^/ERS^lo^ tumours and MKS^hi^/ERS^hi^ tumours with RD, with rates of 61% and 93%, respectively. The poor prognosis of MKS^hi^/ERS^lo^ tumours was also confirmed in TCGA, where they exhibited the worst overall survival among ER+/HER2− breast cancers. The worse prognosis and increased endocrine resistance of MKS^hi^/ERS^lo^ tumours cannot be explained by different ER expression in immunohistochemistry, as the ER expression of these tumours in TCGA was not significantly different from that of MKS^hi^/ERS^hi^ tumours (Supplementary Fig. S6). Thus, novel treatment strategies are needed to improve survival for these patients.

Adjuvant abemaciclib and ribociclib added to standard-of-care endocrine therapy were recently shown to improve recurrence-free survival in ER+/HER2− clinically high-risk breast cancers [33, 34]. However, if the benefit extends equally to all molecular subsets of ER+/HER2− breast cancers remains unknown. We investigated whether MKS^hi^/ERS^lo^ tumours displayed molecular features predictive of response to CDK4/6i therapy. The Rb loss-of-function signature (RBsig) derived by Malorni et al. has been shown to discriminate palbociclib-resistant versus sensitive breast cancer cell lines [23] and to be enriched in palbociclib-resistant tumours of patients treated in the NeoPalAna trial [24]. The Interferon-Related Palbociclib-Resistance Signature (IRPS) is a gene signature capturing different biological pathways and providing prognostic information independent to RBsig that has been shown to be correlated with resistance to CDK4/6i in both the NeoPalAna and neoMONARCH trials [24]. High expression levels of *CCNE1*, the gene encoding for cyclin E, were associated with resistance to palbociclib in three independent clinical trials: the PALOMA-3 study and the neoadjuvant NeoPalAna and POP studies [16, 25]. We observed that both RBsig and CCNE1 predicted low sensitivity to CDK4/6i in MKS^hi^/ERS^lo^ tumours, whereas the IRPS did not provide informative results. These findings were consistent with our analysis of samples from the NeoPalAna study, where MKS^hi^/ERS^lo^ tumours displayed persistently high proliferation levels on Day 15 of neoadjuvant endocrine therapy plus palbociclib, indicating poor response to the doublet therapy.

These results raise the possibility that MKS^hi^/ERS^lo^ tumours may not derive optimal benefit from adjuvant CDK4/6i therapy. However, it should be noted that generalizing these findings to other CDK4/6i may not be justified, as different CDK4/6i have shown varying efficacy in different clinical settings. Indeed, abemaciclib has demonstrated single-agent activity in endocrine-resistant metastatic breast cancer [35], and abemaclib and riboclib improved overall survival in the metastatic setting and decreased invasive recurrences in stage IIB/III cancers [33, 34], while palbociclib did not. Therefore, the potential role of abemaciclib or ribociclib in improving the poor outcome of MKS^hi^/ERS^lo^ tumours cannot be excluded.

We also found that MKS^hi^/ERS^lo^ tumours had high rates of *TP53* gene mutations, approximately twice the rate seen in ER+ tumours in general [36]. *TP53* mutations in ER+/HER2− breast cancers are known to be associated with poor prognosis and resistance to endocrine therapy [37], and TP53-mutated tumours more frequently have high Oncotype DX recurrence score results than wild-type tumours [38]. Additionally, we found that PIK3CA mutations were present in about one-third of MKS^hi^/ERS^lo^ tumours, indicating a potential therapeutic option with PI3Kα-specific inhibitors such as alpelisib in this subgroup.

Initial studies including all ER+/HER2− breast cancers concluded that these tumours are immune infiltration poor, or “immunologically cold”, and have lower TMB compared to other breast cancer subtypes [39, 40]. However, more recent analyses have indicated that a subset of ER+/HER2− breast cancers have high tumour infiltration and immune cell activation similar to those seen in TNBC [41]. Furthermore, accumulating clinical trial evidence suggests a potential benefit of immunotherapy in a subset of ER+/HER2− tumours. Pembrolizumab monotherapy has been shown to be effective in a small cohort of heavily pretreated patients with ER+/HER2− metastatic breast cancer [42] and demonstrated meaningful antitumor activity in cancers with high TMB, including ER+/HER2− breast cancers [43]. Moreover, the addition of pembrolizumab to neoadjuvant chemotherapy has significantly improved the pCR rate in MammaPrint ultrahigh (MP2) ER+/HER2− breast cancers in the I-SPY2 trial [17]. The benefit from ICI therapy in ER+/HER2− MP2 breast cancers was further demonstrated in the durvalumab and olaparib arm of the I-SPY2 trial, where the pCR rate improved from 22% with standard-of-care chemotherapy to 64% with the addition of durvalumab and olaparib [31]. We evaluated potential biomarkers of immunotherapy benefit in MKS^hi^/ERS^lo^ tumours to determine whether these tumours might represent the subgroup that could benefit from ICI therapy. Although MammaPrint results were not available in our patient cohorts, we found that MKS^hi^/ERS^lo^ tumours have the highest TMB among ER+/HER2− cancers, with a mutation load similar to that of TNBC. In MKS^hi^ cancers, TMB inversely correlated with ERS, which aligns with previous studies indicating a twofold higher mutation rate in AI-resistant compared to AI-sensitive ER+/HER2− breast cancers [44].

The high TMB observed in MKS^hi^ tumours provides a possible mechanistic explanation for their endocrine resistance and increased immunogenicity, as indicated by the elevated expression of TIL and T-cell inflamed metagenes, both associated with a greater benefit from ICI therapy [45].

Our findings are also consistent with earlier reports that demonstrated that ER+/HER2− tumours with high TILs often exhibit grade 3, lower ER expression, higher Ki67 expression and high recurrence scores [46, 47]. The high expression of immune markers in MKS^hi^/ERS^lo^ tumours, along with the inverse relationship between ER-related genes and immune gene signatures, suggests that the least endocrine-sensitive cancers may be the most responsive to immunotherapy. Others have also shown that endocrine-resistant luminal B tumours display upregulation of the INF-γ signalling pathway [48]. Our analysis of ER+/HER2− tumours included in the pembrolizumab arm and corresponding controls in the I-SPY2 trial, which specifically focused on MammaPrint-high tumours, supports this hypothesis.

We observed that in the ERS^hi^ group, the pCR rates were approximately 10% to 12% in both treatment arms, while in the ERS^lo^ group, the pCR rate was significantly higher with pembrolizumab than with chemotherapy alone, with rates of approximately 42% versus 21%, respectively. However, likely due to the small sample size, the interaction test between ERS and treatment with chemotherapy plus pembrolizumab was not statistically significant. Results from the GIADA trial also suggest that ER+/HER2− breast cancers with an immune-activated state and downregulation of hormone receptor pathways respond favourably to sequential chemotherapy and anti–PD–1 therapy [49]. Nonetheless, the absence of baseline characteristics for the patients included in this and the other datasets we utilized hinders our ability to evaluate how these variables might have influenced our results.

In summary, our findings demonstrate that MKS^hi^/ERS^lo^ tumours represent a distinct molecular subset within ER+/HER2− breast cancers. These tumours are characterized by frequent TP53 mutation, high proliferation, low expression of ER-related genes, and elevated expression of immune metagenes and markers of CDK4/6i resistance. These molecular characteristics provide an explanation for the higher rate of endocrine resistance and overall poorer prognosis observed in these tumours. Importantly, a subset of MKS^hi^/ERS^lo^ tumours exhibits high chemotherapy sensitivity, as indicated by the relatively high pCR rate of approximately 20%, which appears to be further improved by the addition of pembrolizumab to neoadjuvant chemotherapy. Additionally, around 30% of these cancers harbour *PIK3CA* mutations, suggesting that adjuvant PIK3CA inhibitors may represent a class of drugs worth investigating in prospective clinical trials. The benefit from immunotherapy is also based on small studies and will need independent confirmation in larger randomized trials.

### Supplementary information

Supplementary Table 1

Supplementary Table 2

Supplementary figures

## Data Availability

The data analyzed in this study were obtained from the TCGA public access portal (http://cancergenome.nih.gov/) and from Gene Expression Omnibus (GEO) at GSE25066, GSE105777, GSE126870, GSE93204 and GSE194040.
