# Supplementary material for: Breast cancers with high proliferation and low ER-related signalling have poor prognosis and unique molecular features with implications for therapy
Source: Br J Cancer. 2023 Nov 7;129(12):2025–33. doi: 10.1038/s41416-023-02477-7 (PMC10703787; doi:10.1038/s41416-023-02477-7)
Supplement: Supplementary file 1 — Supplementary Table 1 [file 41416_2023_2477_MOESM1_ESM.docx]

| **Characteristic** | | **MKS^hi^/ERS^hi^**  **(n = 68)** | **MKS^hi^/ERS^low^**  **(n = 131)** |
| --- | --- | --- | --- |
| Age | |  |  |
|  | Median | 48,5 | 49 |
| Nodal status - no. (%) | |  |  |
|  | Positive | 24 (35.3) | 39 (29.8) |
|  | Negative | 44 (64.7) | 92 (70.2) |
| T stage - no. (%) | |  |  |
|  | 0 | 0 (0) | 2 (1.5) |
|  | 1 | 4 (5.9) | 3 (2.3) |
|  | 2 | 44 (64.7) | 53 (40.5) |
|  | 3 | 13 (19.1) | 45 (34.3) |
|  | 4 | 7 (10.3) | 28 (21.4) |
| Grade - no. (%) | |  |  |
|  | 1 | 6 (8.8) | 6 (4.6) |
|  | 2 | 41 (60.3) | 49 (37.4) |
|  | 3 | 18 (26.5) | 64 (48.9) |
|  | Unknown | 3 (4.4) | 12 (9.1) |
| PR status - no. (%) | |  |  |
|  | Positive | 62 (91.1) | 80 (61.1) |
|  | Negative | 5 (7.4) | 48 (36.7) |
|  | Indeterminate | 1 (1.5) | 3 (2.2) |

**Supplementary Table 1. Patients’ baseline characteristics In the MDACC dataset**
