# Supplementary material for: Breast cancers with high proliferation and low ER-related signalling have poor prognosis and unique molecular features with implications for therapy
Source: Br J Cancer. 2023 Nov 7;129(12):2025–33. doi: 10.1038/s41416-023-02477-7 (PMC10703787; doi:10.1038/s41416-023-02477-7)
Supplement: Supplementary file 2 — Supplementary Table 2 [file 41416_2023_2477_MOESM2_ESM.pdf]

Supplementary Table 2. Frequency of recurrent genomic alterations in ER+/HER2- tumors stratified according to MKS and ERS status in TCGA

Mutation	Overall (n = 640)	MKS^{lo}/ERS^{hi} (n = 209)	MKS^{lo}/ERS^{lo} (n = 129)	MKS^{hi}/ERS^{hi} (n = 146)	MKS^{hi}/ERS^{lo} (n = 156)	p-value
**PIK3CA**	39%	52%	33%	32%	33%	6.47×10^{-5}
**TP53**	17%	6%	12%	16%	34%	8.28×10^{-11}
**CDH1**	15%	19%	19%	9%	11%	0.013
**GATA3**	13%	9%	16%	14%	15%	0.150
**MAP3K1**	9%	15%	5%	11%	4%	5.85×10^{-4}
**MLL3**	7%	12%	3%	8%	4%	0.0091
**MUC12**	5%	4%	7%	3%	6%	0.401
**MAP2K4**	5%	5%	2%	8%	3%	0.119
**NCOR1**	4%	5%	7%	3%	3%	0.315
**SPEN**	4%	5%	5%	3%	3%	0.733
**MUC4**	4%	1%	7%	5%	4%	0.057
**PTEN**	4%	5%	5%	1%	5%	0.088
**DMD**	4%	2%	2%	3%	8%	0.054
**RUNX1**	4%	4%	7%	3%	1%	0.081
**TBX3**	3%	4%	2%	5%	2%	0.196
