# Supplementary material for: Breast cancers with high proliferation and low ER-related signalling have poor prognosis and unique molecular features with implications for therapy
Source: Br J Cancer. 2023 Nov 7;129(12):2025–33. doi: 10.1038/s41416-023-02477-7 (PMC10703787; doi:10.1038/s41416-023-02477-7)

Supplementary Figure 1. Clinical and pathological outcomes of patients treated with neoadjuvant systemic therapy stratified according to MKS and ERS status A, B) PathR rates **(A)** and 4-year distant event-free survival rates **(B)** in patients receiving taxane-anthracycline-based neoadjuvant chemotherapy at MD Anderson Cancer Center. **C, D)** 4-year distant event-free survival rates in patients with MKS ^{hi}/ERS ^{hi} **(C)** and MKS ^{hi}/ERS ^{lo} **(D)** tumors according to pathological response. **E, F)** Ki67 levels **(E)** and MKS expression **(F)** at surgery in patients receiving neaodjuvant AI in the POETIC study.

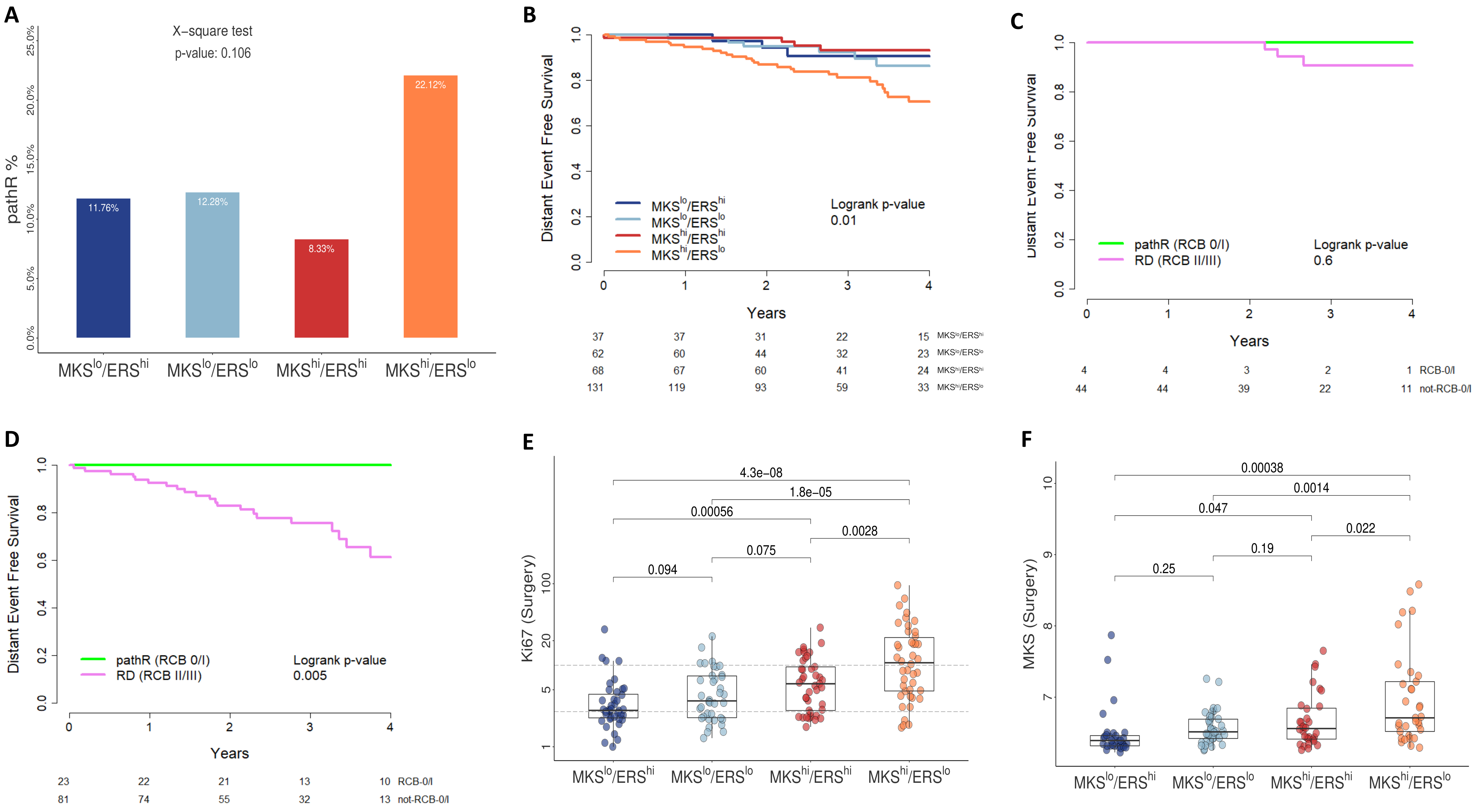

Supplementary Figure 2. Clinical outcomes and genomic alterations of ER+/HER2- tumors stratified according to MKS and ERS status in TCGA

A) 5-year Overall Survival. **B)** Most frequently mutated genes

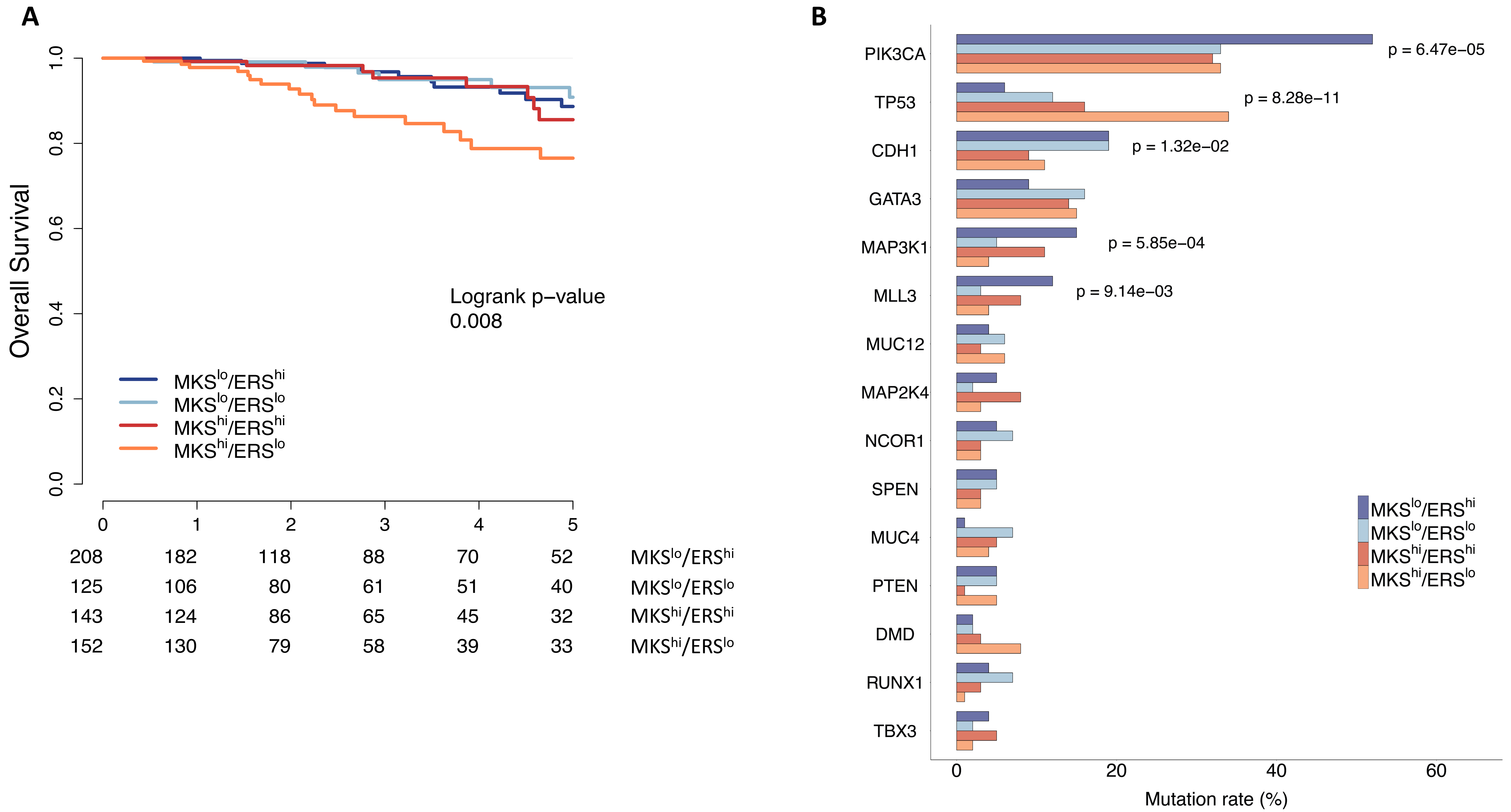

Supplementary Figure 3. Expression of potential predictive biomarkers for CDK4/6 inhibitors in ER+/HER2- tumors stratified according to MKS and ERS status in the TCGA dataset. A) RB signature expression levels. **B)** Interferon-Related Palbociclib-Resistance Signature expression levels. **C)** Cyclin E expression levels

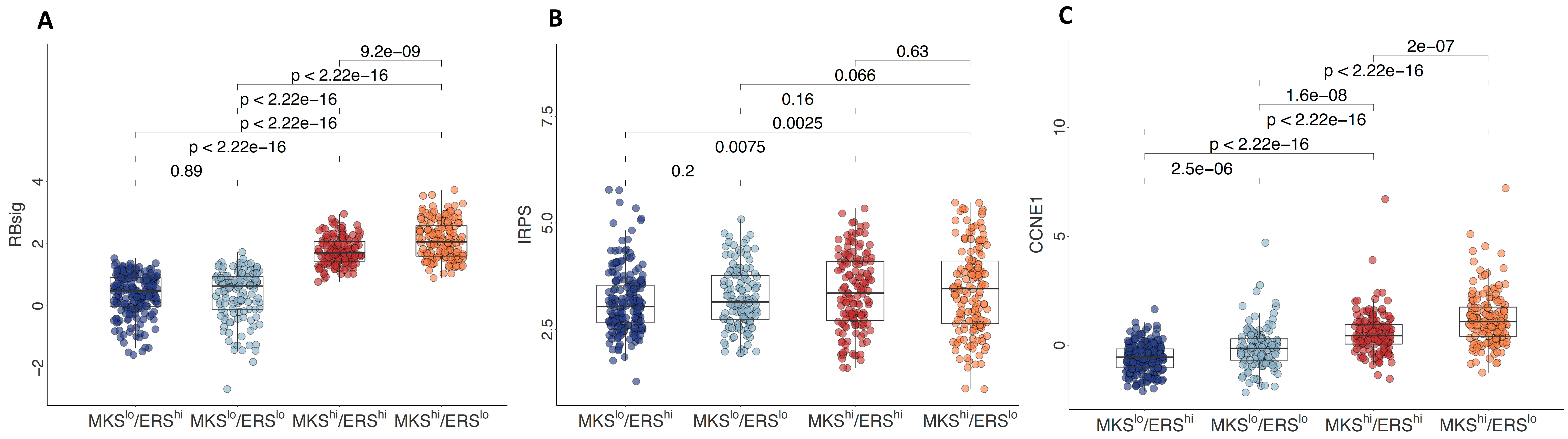

Supplementary Figure 4. Tumor mutational burden (TMB) in breast cancer subgroups in TCGA.
A) TMB in ER+/HER2- tumors stratified according to MKS and ERS status. **B)** TMB in the whole breast cancer cohort stratified according to ER and HER2 status. **C)** TMB in ER+/HER2- tumors stratified according to ERS quartiles. **D)** TMB in MKS ^{hi} tumors stratified according to ERS quartiles. **E, F)** TMB in ER+/HER2- tumors (whole cohort and molecular subgroups) stratified according to PIK3CA **(E)** and TP53 **(F)** mutation status

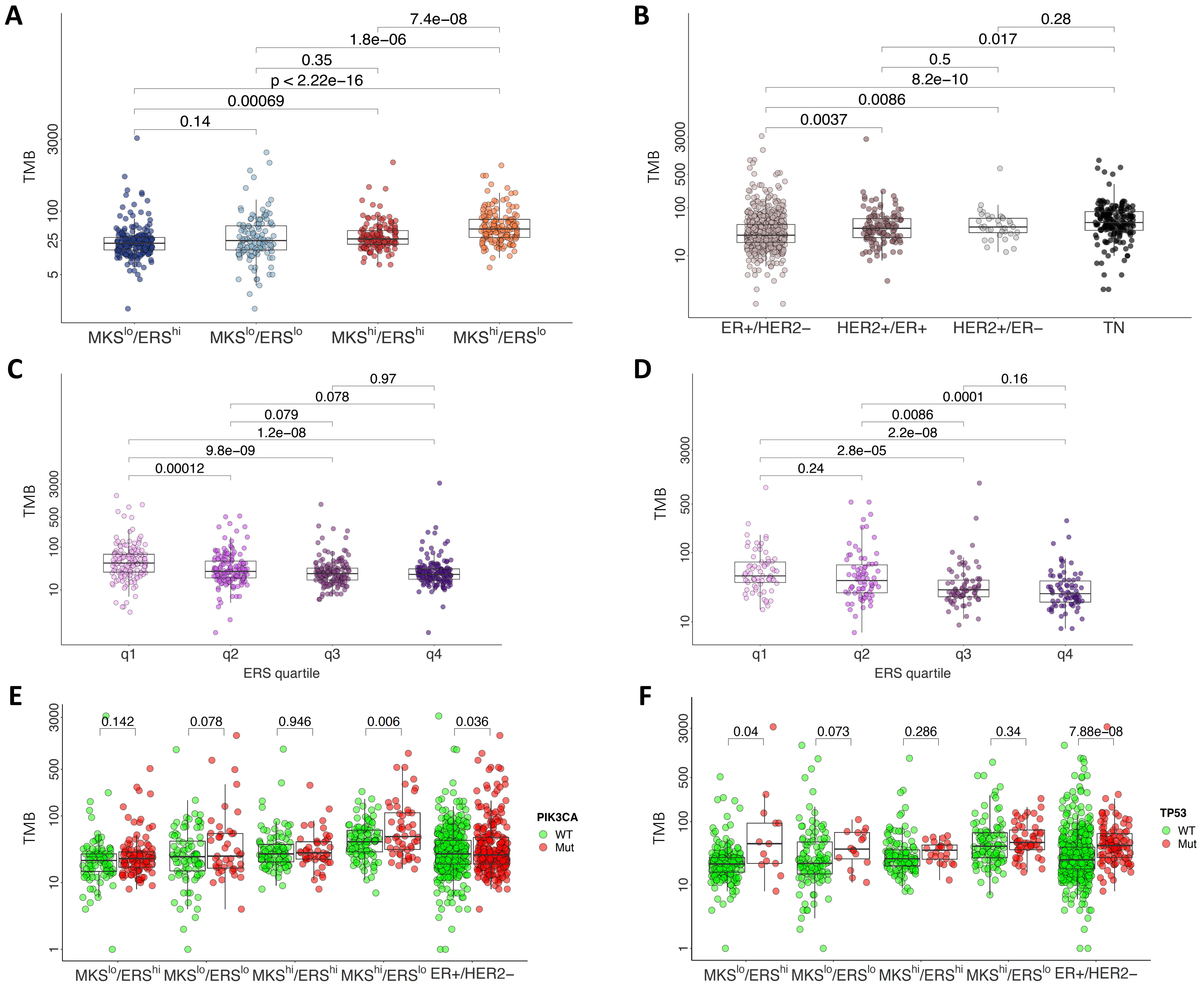

Supplementary Figure 5. Expression of potential predictive biomarkers for immune checkpoint inhibitors in ER+/HER2- tumors in TCGA dataset. TILs metagene **(A, B, C)** and T cell-inflamed GEP **(D, E, F)** expression levels in ER+/HER2- tumors stratified according to MKS and ERS status **(A and C)**, in ER+/HER2- tumors stratified according to ERS quartiles **(B and D)**, in MKS^{hi} tumors stratified according to ERS quartiles **(C and F)**

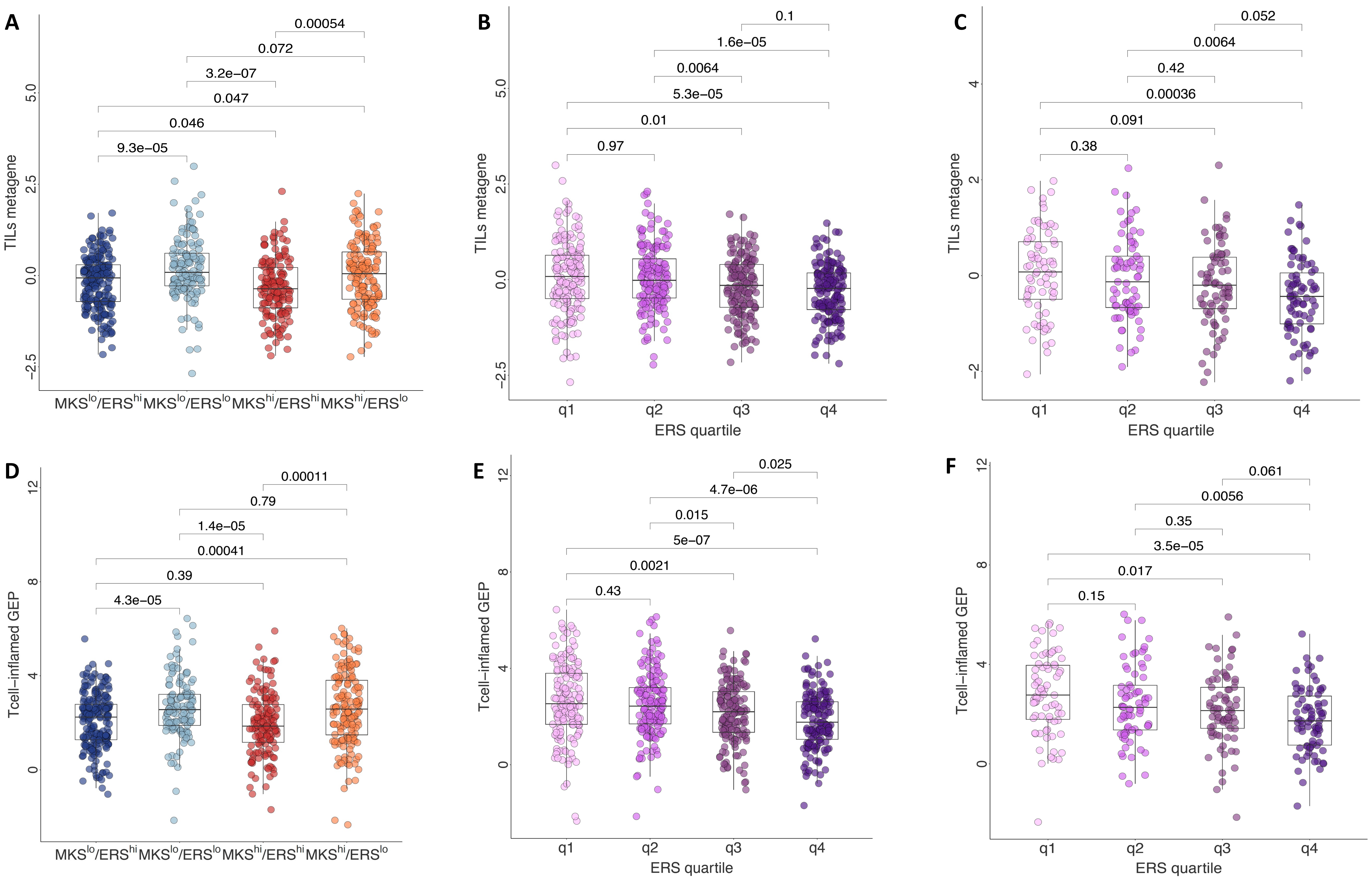

Supplementary Figure 6. Distribution of ER (A) and PgR (B) expression levels by IHC within MKS^{hi}/ERS^{hi} (red bars) and MKS^{hi}/ERS^{lo} (orange bars) tumors in TCGA

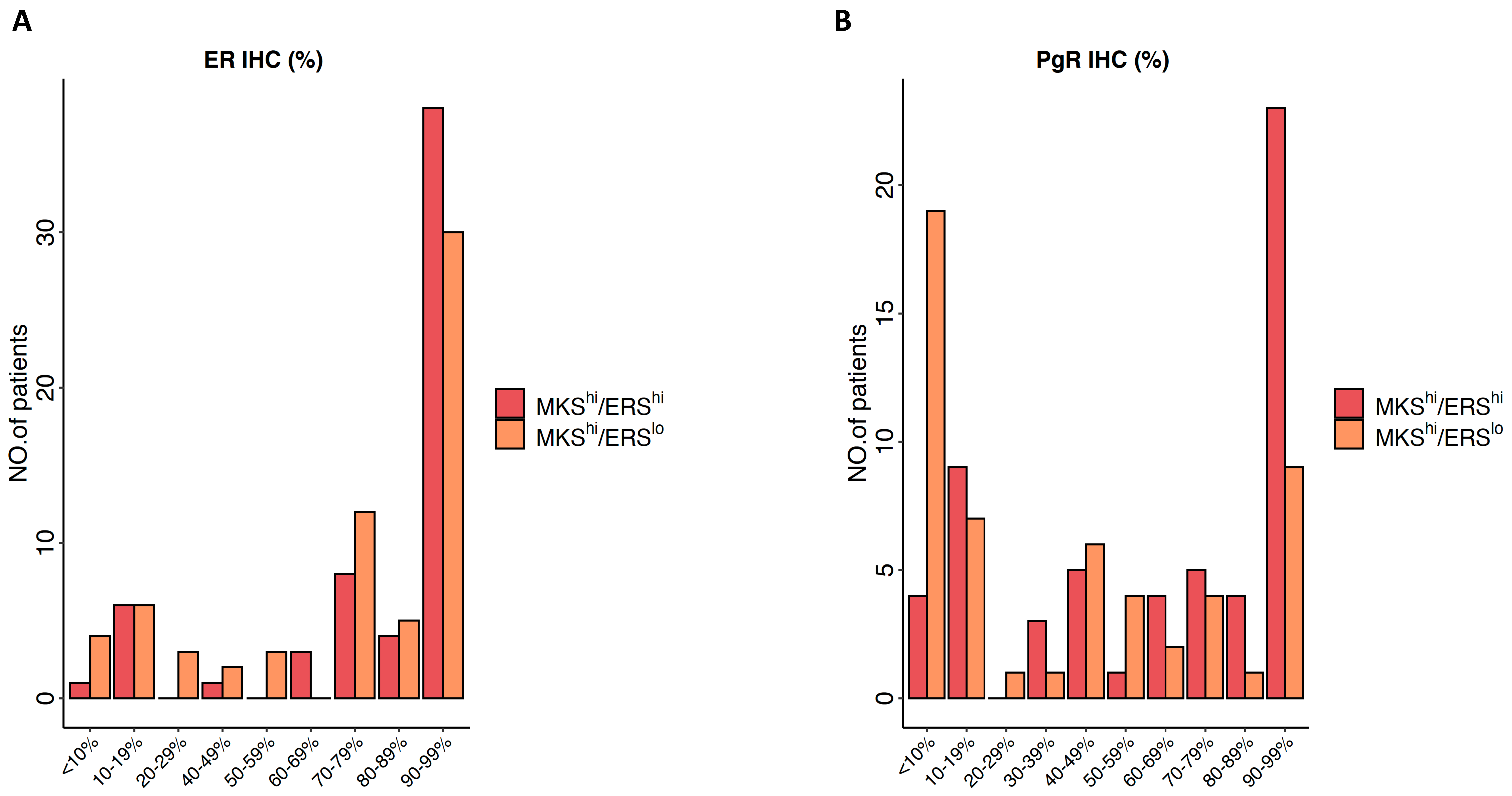

Supplement: Supplementary file 3 — Supplementary figures [file 41416_2023_2477_MOESM3_ESM.pdf]
